# Recent Advances and Trends in the Dairy Field

**DOI:** 10.3390/foods11131956

**Published:** 2022-07-01

**Authors:** Jordi Saldo, Esther Sendra

**Affiliations:** 1 Centre d’Innovació, Recerca i Transferència en Tecnologia dels Aliments (CIRTTA), Animal and Food Science Department, Facultat de Veterinària, Edifici V. Campus de la UAB, 08193 Bellaterra, Barcelona, Spain; jordi.saldo@uab.cat; 2 Centro de Investigación e Innovación Agroalimentaria y Agroambiental (CIAGRO-UMH), Miguel Hernández University, Carretera de Beniel, km 3.2, 03312 Orihuela, Alicante, Spain

Dairy products have been an important part of the human diet for most societies since the Neolithic period. Despite adherence to traditional products such as traditional cheese varieties, which are considered highly prestigious, being important, innovations in dairy products continue. Dairy science is among the oldest fields of food science and technology. Many universities have dairy science sections or departments both in their animal or food science faculties or schools, and several scientific journals specialize in dairy science and technology. Consequently, a huge amount of scientific research in this field is available. A search in Scopus for the term “Dairy” yielded more than 638,250 entries published from 1855 to 2022. The first registered study on this database dates back to 1863 and was published in the *British Medical Journal* [1]. This study and most studies during the first 50 years of scientific publications on dairy science focused on animal health and food safety issues. One of the first papers published on dairy foods was a manuscript about condensed milk [2], published in *Nature* in 1873. In the late XIXth and early XXth centuries, several other publications providing methodologies for analysing dairy foods [3,4,5] appeared. Dairy science has continuously evolved since then, and in recent years, scientific advances and production have increased dramatically, mainly as state-of-the-art molecular methods become increasingly available. The application of novel processing technologies requires a deeper knowledge of the structure and functionality of milk proteins, allowing for the development of novel products with improved functional and healthy properties.

This Special Issue contains a collection of reviews prepared by highly recognized scientists in the dairy science field. In this editorial, we aim to summarize the highlights from these reviews. 

Fermented milk products such as cheese and yogurt are among the oldest systems used for milk preservation, which also serve to enhance its nutritional quality. Traditional dairy products have been investigated in depth due to the availability of new analytical procedures. Mayo et al. [6] reviewed the knowledge on microbial communities and their interactions in cheese. Their study provides insights and state-of-the-art knowledge on common starter and non-starter lactic acid bacteria (LAB), main cheese types, available culture-independent molecular methods for the study of microbial populations, cheese microbiota, the microbial interaction in cheese, and the dynamics of microbial interaction in cheese and microbiota-based starters. Their review points to the need for more knowledge to be able to predict cheese fermentations, to avoid technological failures, and to develop strain mixtures as improved starter cultures for improving cheese quality and safety. Chemical or microbial biomarkers need to be defined, and omics and computational developments need to be applied to study microbial interactions and the molecular basis behind them in the cheese ecosystem. Mayo et al. defined a three-step scenario: a model system for studying the mechanisms of interaction in simple microbial communities; confirmation of the mechanisms at the cheese ecosystem scale; and finally, once the microbial interactions and the effect of biotic and abiotic factors are understood, the design of model microbial communities that are able to mimic desirable cheese fermentation processes and sensory properties.

Dapkevicius et al. [7] presented the current trends of enterococci in dairy products, focusing on both beneficial and deleterious properties. Enterococci are commonly found in artisanal cheeses, affect cheese sensory properties, modulate cheese microbiota, and help control pathogenic populations. They have the ability to easily adapt to adverse environmental conditions; however, they can also become opportunistic pathogens due to the plasticity of their genome and their propensity to trade genes. They can be useful in cheese as adjunct, protective, or even probiotic bacteria, but they can also carry virulence factors. The review by Dapkevicius et al. focuses on the role of enterococci in artisanal cheeses and their potentialities. Enterococci became a genus in 1984; they were previously classified as streptococci from group D of Lancefield. This genus is associated with the human intestinal environment as a habitat, but enterococci can easily colonize different ecological habitats and can enter a viable non-culturable state to resist harsh environmental conditions. Enterococci are commonly present in raw milk and in milking environments. Up to nineteen species have been isolated in artisanal cheeses so far, mainly due to the availability of culture-independent methodologies. They mainly contribute as a non-starter LAB in flavour development or as a protective culture, whereas only a few species have bene reported to have good acidification abilities. Their technological properties include flavour enhancements; uneven proteolytic activity (including some species producing biogenic amines); the prevention of bitterness and related flavour defects from developing; the production of exopolysaccharides; lipolytic activity; and the ability to metabolize citrate with the release of low molecular weight volatiles, the formation of eyes in cheeses, and the release of bacteriocins. Multiple potential health benefits have been related to the enterococci species, including the release of bioactive peptides (antihypertensive, antimicrobial, anticholesterolemic, and antihyperglycemic); the production of conjugated linoleic acid; and the production of the neurotransmitters serotonin and γ-aminobutyric acid (GABA), which affect the gut–brain–axis. However, much more data are needed to support those health claims and to ensure safe consumption of species of this genus. The pathogenic activities associated with enterococci are mainly nosocomial infections, with enterococci acting as opportunistic pathogens. Virulence factors and antibiotic resistance to different antibiotics were reviewed by Dapkevicius et al., who pointed to the need for further studies applying omics technologies to enterococci in artisanal cheeses to gain a better understanding of their technological and health effects as well as to reduce their potential deleterious properties.

One field related to dairy science that is gaining the attention of consumers and companies are plant-based alternatives to dairy products; these alternatives are not dairy but take the nutritional and sensory qualities of dairy products as a reference. Montemurro et al. [8] reviewed plant-based alternatives to yogurt and the challenges faced by this sector. Plant-based yogurt-like products face major challenges when imitating the nutritional, sensory, functional, and technological (texture and shelf-life) properties of yogurts. The main constraints of raw vegetable materials include their low protein contents; their coagulation properties, which are different from those of caseins; the need for additives to provide texture (with the current market showing a clear trend towards reducing the number of additives for clean labelling); the need to maintain stability during shelf-life; and the presence of antinutritional factors. The manuscript by Montemurro et al. focuses on the main ingredients, starter cultures, processing conditions, and texturing agents used in the commercial and experimental development of plant-based yogurt-type products. Cereals, pseudocereals, legumes, and nuts are used as raw materials, and their potential advantages and limitations are reviewed together with possible biotechnology-based solutions to those challenges. As an example, exopolysaccharide-producing lactic acid bacteria starter cultures may be used to develop desirable textures and to avoid the use of or to reduce the number of additives. The nutritional and functional aspects of plant-based products are also reviewed. Commercial plant-based products have highly variable nutritional profiles. From the nutritional point of view and to provide high-quality proteins similar to those of dairy products, plant-based alternatives need to be carefully formulated (combining raw materials: cereals and legumes). Additionally, the presence of antinutritional factors needs to be reduced. From the technological and food safety points of view, fast acidification cultures are needed to develop a low pH within an appropriate fermentation time. Some plant-based products are valuable sources of fibres and phenolic compounds. Sensory acceptability and stability during refrigerated shelf-life are also major challenges in the improvement of these products. Researchers and companies are working intensively to improve those products to meet the great demand from consumers, and new advances in this field are expected.

The functional properties of milk proteins are dependent on their structural organizations, and this structure can be studied using different methods for globular whey proteins, with caseins being more elusive due to their lack of tertiary or even secondary structures. Nuclear magnetic resonance (NMR) is able to elucidate the characteristics of caseins through the study of phosphoserine and its relationship with colloidal calcium phosphate. This topic was reviewed by Markoska et al. [9], who paid attention to both whey proteins and caseins. Within milk proteins, β-lactoglobulin has become the most extensively studied due to its availability and abundance. α-lactalbumin has been studied in detail, especially for its denaturing and refolding processes, and has become a model for NMR studies. The effects of different processing conditions applied to milk can have an important impact on the organisation of caseins within the micelle and even its partition between the micellar and serum phases of β-casein. Caseins are less studied milk proteins in terms of NMR. The regions of greater interest are those with phosphorylated serine due to their relevance in the stabilisation of casein micelles through calcium. In casein αs1, we have one of these regions between amino acids 59 and 79, and a total of eight phosphorylated residues, making it sensitive to calcium levels. Using NMR, the N-terminus region has also been studied for its tendency to self-associate. Its few secondary structures remain mostly invariable irrespective of temperature or the presence of divalent cations. In casein αs2, we have two phosphorylated regions between amino acids 1 and 21 and 46–70, and a total of 10–13 phosphorylated serine residues, making it the most calcium-sensitive protein. Due to its low abundance and the difficulty in isolating it, this protein has been scarcely studied. The phosphorylated regions have a helical structure, while the ones farther from the N-terminus suffer distinctive changes in their conformation as a response to calcium concentration. β-casein has its phosphorylated region between amino acids 1 and 25. It is predicted to have no or few secondary structures. Its N-terminus is the most hydrophobic part of the molecule and, due to that, has received the most attention. κ-casein is relevant for its hydrophilic C-terminus, which contributes to its characteristic of populating the surface of the micelle. Its structure is extremely random in all caseins. On top of the study of individual caseins, the micelle as a whole has been studied by NMR under the influences of pH and temperature. One of the points of great interest has been the characterisation of calcium phosphate nanoclusters and the P-Ca bonds that stabilise the micelles.

Milk thermal treatment has been used for decades to ensure its safety and to extend its shelf-life. These thermal treatments have experienced relevant advances to better preserve the characteristics of the original raw product. To improve the retention of the flavour and technological properties of raw milk, some non-thermal technologies have also been developed. One of these technologies is microfiltration, which can physically remove bacterial cells from skimmed milk without high temperatures and with moderate pressures. This technology that separates the particles according to their size can also fractionate casein micelles from whey proteins using ultrafiltration membranes. Conducting the microfiltration process at warm temperatures decreases the viscosity and improves the transfer properties at the cost of microbial quality and some issues with fouling, so cold microfiltration has been studied as an alternative [10]. All of the relevant conditions that can affect the performance of microfiltration were reviewed by France et al. [10]. The differences between warm and cold filtration received special attention in this review, in particular the effects on microbiology and fouling. The changes in mineral solubility and the change in casein micelle size with the concomitant release of some β-caseins to the serum fraction have a relevant impact not only on fouling but also on the partition of the different milk constituents passing through the membrane. Novel fractions, such as β-casein-enriched fractions, can be produced using cold microfiltration, opening the opportunity to develop innovative dairy products based on these novel fractions.

Milk from different mammal species possess different properties not only due to their fat and protein contents and to the different proportions of their proteins but also due to the difference in the sequences of whey and casein proteins. The variability within bovine genotypes is also a source of diversity in milk proteins. This topic was reviewed by Gai et al. [11]. The variability in β-lactoglobulin was studied the earliest back in 1957, followed by studies on caseins for their relevance to dairy technology. The oldest studies characterized the different forms of the protein for their electrophoretic mobility under defined conditions. The genetic variability can be expressed on just one type of protein when the character is homozygotic or when there is complete dominance, but when a co-dominance exists, heterozygotic cows can express two variants of the same protein. Some of this variability has been invisible to breeders, so a large number of polymorphisms occur in different populations. However, the variant A4 of β-casein has only been identified in a Korean cattle breed and the variant E was found in Piemonte cattle. The most common variant of β-casein is the A2 variant, probably selected unwittingly by generations of breeders due to its association with higher yields, but most of these populations are heterozygotic. Small changes in the peptide sequence can be relevant to the technological properties of milk in the production of firmer curds during cheesemaking when variant C of casein αs1 is present, compared with variant B, although variant B is predominant. Variants A1 and B of β-casein produce a peptide that is released through digestion, known as β-casomorphin, while variant A2 does not lead to the release of this particular peptide. β-casomorphin has been controversially reported to be associated with health problems of a different kind, and it is presumed that A2 milk is more beneficial for human health. The coagulation properties can be also affected by the variability among milk proteins, mainly because of their effects on the hydrophobicity or phosphorylation level of individual caseins, which also affect casein micelle size. This knowledge of the technological properties of thermal stability, coagulation and emulsification, and foaming and their relationships with the genetic composition of cows will strengthen the coordination between animal production and dairy technology, as breeders can now know the genetic composition of cows and bulls to produce calves with the desired traits for each specific dairy product.

This work is highly relevant for other researchers and academia as it provides cutting-edge knowledge on the topics reviewed in a summarized and understandable manner. The editors thank all authors for their contributions to this Special Issue as well as for their work and long-term commitment to advancements in dairy science.
